# Natriuretic peptide receptors regulate cytoprotective effects in a human *ex vivo* 3D/bioreactor model

**DOI:** 10.1186/ar4253

**Published:** 2013-07-24

**Authors:** Nicholas Peake, Nyan Su, Manoj Ramachandran, Pramod Achan, Donald M Salter, Dan L Bader, Amie J Moyes, Adrian J Hobbs, Tina T Chowdhury

**Affiliations:** 1 School of Engineering and Materials Science, Queen Mary University of London, Mile End Road, London E1 4NS, UK; 2 Department of Orthopaedics and Trauma, The Royal London Hospital and Barts & The London School of Medicine & Dentistry, Queen Mary University of London, Whitechapel Road, London E1 1BB, UK; 3 Centre for Molecular Medicine, MRC Institute of Genetics and Molecular Medicine, University of Edinburgh, Crew Road, Edinburgh EH4 2XU, UK; 4 Faculty of Health Sciences, University of Southampton, Southampton General Hospital, Southampton SO16 6YD, UK; 5 William Harvey Heart Centre, Barts and The London School of Medicine and Dentistry, Queen Mary University of London, Charterhouse Square, London EC1M 6BQ, UK

## Abstract

**Introduction:**

The present study examined the effect of C-type natriuretic peptide (CNP) and biomechanical signals on anabolic and catabolic activities in chondrocyte/agarose constructs.

**Methods:**

Natriuretic peptide (Npr) 2 and 3 expression were compared in non-diseased (grade 0/1) and diseased (grade IV) human cartilage by immunofluoresence microscopy and western blotting. In separate experiments, constructs were cultured under free-swelling conditions or subjected to dynamic compression with CNP, interleukin-1β (IL-1β), the Npr2 antagonist P19 or the Npr3 agonist cANF^4-23^. Nitric oxide (NO) production, prostaglandin E_2 _(PGE_2_) release, glycosaminoglycan (GAG) synthesis and CNP concentration were quantified using biochemical assays. Gene expression of Npr2, Npr3, CNP, aggrecan and collagen type II were assessed by real-time qPCR. Two-way ANOVA and a *post hoc *Bonferroni-corrected *t*-test were used to analyse the data.

**Results:**

The present study demonstrates increased expression of natriuretic peptide receptors in diseased or older cartilage (age 70) when compared to non-diseased tissue (age 60) which showed minimal expression. There was strong parallelism in the actions of CNP on cGMP induction resulting in enhanced GAG synthesis and reduction of NO and PGE_2 _release induced by IL-1β. Inhibition of Npr2 with P19 maintained catabolic activities whilst specific agonism of Npr3 with cANF^4-23 ^had the opposite effect and reduced NO and PGE_2 _release. Co-stimulation with CNP and dynamic compression enhanced anabolic activities and inhibited catabolic effects induced by IL-1β. The presence of CNP and the Npr2 antagonist abolished the anabolic response to mechanical loading and prevented loading-induced inhibition of NO and PGE_2 _release. In contrast, the presence of the Npr3 agonist had the opposite effect and increased GAG synthesis and cGMP levels in response to mechanical loading and reduced NO and PGE_2 _release comparable to control samples. In addition, CNP concentration and natriuretic peptide receptor expression were increased with dynamic compression.

**Conclusions:**

Mechanical loading mediates endogenous CNP release leading to increased natriuretic peptide signalling. The loading-induced CNP/Npr2/cGMP signalling route mediates anabolic events and prevents catabolic activities induced by IL-1β. The CNP pathway therefore represents a potentially chondroprotective intervention for patients with OA, particularly when combined with physiotherapeutic approaches to stimulate biomechanical signals.

## Introduction

There is an urgent demand for long-term solutions to improve osteoarthritis (OA) treatment in the ageing population. There are drugs that control the pain but none that stop the progression of the disease in a safe and efficient way. More effective intervention, augmented by early diagnosis and integrated biophysical therapies are therefore needed. Unfortunately, progress has been slow due to the wide variety of experimental models that examine the effect of mechanical stimuli and chondroprotective agents on signal transduction pathways. Accordingly, our understanding of the early mechanopathophysiology is poor, particularly the way in which mechanical stimuli influence cell function and regulate matrix synthesis. This makes it difficult to identify reliable targets and design new therapies for OA treatment.

Growing evidence suggests that stimulation of the C-type natriuretic peptide (CNP) signalling pathway may contribute to anabolic events and potentially provide a new therapeutic application for conditions with loss of cartilage matrix. For example, treatment with CNP has been reported to increase both collagen and proteoglycan synthesis and to enhance cell proliferation in chondrocytes cultured in monolayer or pellet culture [1,2]. In an *ex vivo *human chondrocyte three-dimensional (3D)/bioreactor model, we showed increased cell proliferation and proteoglycan synthesis, and suppression of catabolic activities in response to CNP [3]. Indeed, in our previous study, exogenous CNP was found to be protective and mediates enhanced cell proliferation and extracellular matrix synthesis via 3, 5-cyclic guanosine monophosphate (cGMP)-dependent protein kinase II (PKGII). Furthermore, the protective effects of CNP were enhanced with stimulation by mechanical loading in human chondrocyte/agarose constructs cultured with IL-1β. However, the interplay of CNP and biomechanical signals in IL-1β-treated chondrocytes has not been examined in detail. In a previous study, the natriuretic peptide receptor (Npr)2guanylyl cyclase B and -cGMP (Npr2/cGMP) pathway was shown to mediate increased cell proliferation in rat chondrocytes treated with CNP [4]. In this model, upregulation of the Npr2cGMP system by CNP is essential for cartilage development and involves PKGII mechanisms in late proliferative and pre-hypertrophic zones of growth-plate cartilage [4-9]. Furthermore, disruption of the genes encoding CNP and PKGII results in impaired growth of endochondral bones and leads to severe dwarfism and skeletal defects [5-7]. Conversely, overexpression of CNP results in skeletal overgrowth and rescued dwarfism in a murine model of human achondroplasia [9]. Taken together, the *in vitro *and genetic studies highlight the importance of CNP signalling in cartilage and bone remodelling, and offers the potential of CNP in the treatment of OA and skeletal diseases.

The molecular mechanisms underpinning CNP regulation of cartilage remodelling remain elusive. CNP binding to Npr2 leads to increased cGMP levels, which modulates the downstream activities of PKGs, cGMP-regulated ion channels (CGi) and cGMP-regulated phosphodiesterase (PDE) subtypes [6,10,11]. In contrast, Npr3 does not possess a guanylyl cyclase domain and until recently, was thought to act as a decoyclearance receptor, thereby regulating CNP signalling [12]. It is now apparent Npr3 mediates several signalling effects in rat osteoblasts and inhibits adenylate cyclase or stimulates the G1 proteinphospholipase C pathway, which acts as a positive regulator of bone [13]. Furthermore, CNP has been shown to mediate a vasoprotective profile via Npr3-dependent extracellular signal-regulated kinase (ERK) 1/2 phosphorylation, resulting in augmented endothelial cell proliferation and inhibition of vascular smooth muscle growth [14]. Characterization of the Npr2 and Npr3 signalling cascade in cartilage has not been investigated previously and a greater understanding of the mechanism underpinning these pathways is needed. We hypothesise that the Npr2 and Npr3 pathways have overlapping protective roles in maintaining cartilage homeostasis and may slow pathogenesis and restore tissue damage *in vivo*. In addition, mechanical loading could interfere with the Npr pathways and augment an anabolic response. The present study therefore examined the relationship between mechanical loading and CNP signalling in detail and determined whether the Npr cascade in combination with mechanical signals demonstrates a chondroprotective profile *in vitro*.

## Materials and methods

### Chondrocyte isolation and culture in agarose constructs

Human cartilage was obtained from twenty patients (age 55 to 75 years), with ethical approval (East London and The City Research Ethics Committee) and informed patient consent, undergoing total knee arthroplasty at the Royal London Hospital, Barts Health NHS Trust, London, UK. Cartilage was removed from the femoral condyles and tibial plateaux. The morphology of the cartilage specimens was graded for gross degenerative changes according to the International Cartilage Repair Society (ICRS) classification, and tissues that represent non-diseased (grade 0 or 1) and early (grade 2) OA were used for experiments. Each experimental condition was repeated with chondrocytes from four to five different donors. Cartilage tissue was diced and incubated on rollers for 1 hour at 37°C in DMEM supplemented with 10% (vol/vol) (FCS) + 2 μ*M *L-glutamine, 5 μg/ml penicillin, 5 μg/ml streptomycin, 20 mM Hepes buffer, and 0.05 mg/ml L-ascorbic acid + 700 unit/ml pronase, and incubated for a further 16 hours at 37°C in DMEM + 10% FCS, supplemented with 100 units/ml collagenase type XI (Sigma-Aldrich, Poole, UK). The cell suspension was washed and viable chondrocytes counted using a hemocytometer and trypan blue. Cells were finally resuspended in medium at a cell concentration of 8 × 10^6 ^cells/ml by using a well-established protocol [15,16]. In brief, the cell suspension was added to an equal volume of molten 6% (wt/vol) agarose type VII in Earle Balanced Salt Solutions (EBSS) to yield a final cell concentration of 4 × 10^6 ^cells/ml in 3% (wt/vol) agarose (Sigma-Aldrich). The chondrocyte/agarose suspension was transferred into a sterile stainless steel mould, containing holes 5 mm in diameter and 5 mm in height and allowed to gel at 4°C for 20 minutes. Constructs were cultured in DMEM + 10% FCS at 37°C in 5% CO_2 _for 24 hours (all from Cambrex Bioscience, Wokingham, UK).

### Effect of pharmacological agents that influence natriuretic peptide signalling in chondrocyte/agarose constructs

The effect of pharmacological agents that influence the natriuretic peptide and cGMP pathway were examined in constructs cultured under free-swelling conditions. This approach allowed us to determine their effects on protein synthesis in the absence of mechanical loading. Constructs were cultured in 1 ml of defined media supplemented with 100 n*M *CNP in the presence and absence of 10 ng/ml IL-1β and/or 5 μM specific sGC antagonist (1H-(1,2,4)oxadiazolo-(4, 3-a)quinoxalin-1-one) (ODQ) and/or 1 μM selective Npr3 agonist cANF^4-23 ^(C-Atrial Natriuretic Factor) (Bachem AG, Bubendorf, Switzerland) and/or 0.5 μM selective Npr2 antagonist cyclic gly-24-ser (P19, Gentaur Molecular Products, Whetstone, UK) for 48 hours [17,18]. At the end of the culture period, the constructs and corresponding media were immediately stored at -20°C before biochemical analysis.

### Application of dynamic compression

In separate experiments, a fully characterized bioreactor compression system (Bose ElectroForce, GillinghamUK) was used to determine the effect of dynamic compression and chemical agents, which influence the natriuretic peptide signalling pathway, on cell metabolism and gene expression in CNP andor IL-1β-treated chondrocyte/agarose constructs. The bioreactor has been extensively described previously [15,16,19]. To review briefly, equilibrated constructs were transferred into individual wells of a 24-well culture plate (Costar, High Wycombe, UK) and mounted within the bioreactor. Media supplemented with 0 or 10 ng/ml IL-1β in the presence and absence of 100 n*M *CNP and/or 0.5 μM P19 and/or 1 μM cANF^4-23 ^was introduced into each well. Strained constructs were subjected to dynamic compression ranging from 0 to 15% strain in a sinusoidal waveform at a frequency of 1 Hz. The compression regimen was applied in an intermittent manner, with a profile of 1.5-hour compression followed by a 4.5-hour unstrained period for both 6- and 48-hour culture periods, as previously described [3]. This resulted in duty cycles equivalent to 5,400 and 43,200 respectively. Control constructs were maintained in an unstrained state within the bioreactor system and cultured for the same time period. At the end of the culture period, the constructs and corresponding media were immediately stored at -70°C before analysis.

### RNA isolation, cDNA synthesis, and real-time quantitative PCR

RNA was isolated from chondrocytes cultured in agarose by using protocols described in the QIAquick Spin gel extraction and RNeasy kits, as previously described (Qiagen, Crawley, West Sussex, UK) [20,21]. By following the manufacturer's instructions, Ambion's DNA-*free *DNase treatment and removal reagents were used to eliminate any contaminating DNA from the RNA sample (Ambion Applied Biosystems, Warrington, UK). RNA was quantified on the Nanodrop ND-1000 spectrophotometer (LabTech, Uckfield, East Sussex, UK), and reverse transcription was performed using the manufacturer's protocols from the Enhanced Avian RT First Strand cDNA synthesis kit, oligo(dT)_23 _primer, and a total of 200 ng of RNA (Sigma Genosys, Cambridge, UK). Real-time quantitative (q)PCR assays coupled with locked nucleic acid (LNA) probes were performed in 25-μl reaction mixtures containing 1 μl cDNA, 12.5 μl JumpStart *Taq *PCR Master Mix, primer pairs, probes detailed in Table 1 and nuclease-free PCR-grade water to 25 μl (Sigma Genosys). Each sample was run in duplicate on the 96-well thermal system of the Mx3000P qPCR instrument (Stratagene, Amsterdam, The Netherlands). Thermocycling conditions comprised an initial polymerase activation step at 95°C for 3 minutes, followed by denaturation of 35 cycles at 95°C for 30 seconds, annealing at 55°C for 1 minute, and extension at 72°C for 1 minute. PCR efficiencies for optimal primer pair and probe concentrations were derived from standard curves (*n *= 3) by preparing a 10-fold serial dilution of cDNA from a sample that represented the untreated control at time-zero conditions. The real-time PCR efficiencies (E) of amplification for each target were defined according to the relation, E = 10^(-1/slope)^. The *R^2 ^*value of the standard curve exceeded 0.9998 and revealed efficiency values ranging from 1.94 to 2.03.

**Table 1:** Description of the LNA probe and primer sequences used to quantify gene expression

Gene	Gene ID	Sequences	Product length
***Npr2***	4882	*Probe: *5'-FAM-ATC**G**CT**G**GC**T**GC**T**TCTATG-BHQ1-3' *Sense: *5'-CCCTTCCCTGATGAACCT -3' *Antisense: *5'-CCCTGCATCTTTTCCACAA -3'	132
***Npr3***	4883	*Probe: *5'-FAM-TTC**T**CT**C**CA**A**AGG**T**TCCCG-BHQ1-3' *Sense: *5' TTTAAGAAAAACTAGTGACATTGGA-3' *Antisense: *5'- CCACCCTTCCTCTTTCCT-3'	146
***CNP***	4880	*Probe: *5-'FAM-CGC**C**AA**T**CT**C**AA**G**GGC-BHQ1-3' *Sense: *5'-TCAGAAGAAGGGCGACAA -3' *Antisense: *5'-TGGCTCCTTTGTATTTGCG -3'	158
***GAPDH***	2597	*Probe: *5'-HEX-CAG**T**CA**G**CC**G**CA**T**CTTCT-BHQ1-3' *Sense: *5'-TCTCTGCTCCTCCTGTTC-3' *Antisense: *5'-CGCCCAATACGACCAAAT-3'	160
***Aggrecan***	176	*Probe: *5-'FAM-CCA**A**CT**C**TT**C**AA**G**GTGA-BHQ1-3' *Sense: *5'-GACTGAAGTTCTTGGAGAA-3' *Antisense: *5'-CACGAAAACCCAGAGTAA-3'	109
***Collagen type II***	1280	*Probe: *5'-FAM-TCT**G**TC**T**CC**T**TG**C**TTGCCA-BHQ1-3' *Sense: *5'-GGAGTCAAGGGTGATCGT-3' *Antisense: *5'-CTTGTGCACCAGCTTCTC-3'	200

Primers used in PCR experiments with locked nucleic acid (LNA) probes produced amplicons between 132 and 160 base pairs. Probes contain fluorescein (FAM) or 6-carboxyhexafluorescein (HEX) as the 5'-reporter dye and Black Hole Quencher 1 (BHQ1) as the 3'-quencher. Nucleotides highlighted in bold denote the LNA base.

Fluorescence data were collected during the annealing stage of amplification, and data were analyzed on the MxPro qPCR software (version 3, Stratagene). Baselines and thresholds were automatically set by the RG-3000 qPCR software and used after manual inspection. The cycle threshold (C_t_) value for each duplicate reaction was expressed as the mean value, and the results were exported into Microsoft Excel for further analysis. The data obtained by PCR assay for glyceraldehyde-3-phosphate dehydrogenase (GAPDH) were validated as a reference gene by displaying the C_t _values as box-and-whisker plots, and the distribution examined under the treatment conditions (data not shown). The C_t _values for *GAPDH *remained stable, with no changes detected under all culture conditions, suggesting its suitability as a reference gene. Relative quantification of *Npr2*, *Npr3*, *CNP*, *aggrecan *and *collagen type II *signals were estimated by normalizing each target to the reference gene, *GAPDH*, and to the calibrator sample (unstrained, untreated control) by a comparative C_t _approach. For each sample, the ratio of target ΔCt and reference ΔCt was calculated, as previously described [20-22].

### Biochemical analysis

The production of nitric oxide (NO) was determined in media by converting nitrate to nitrite by using 1 unit/ml nitrate reductase in 40 μ*M *nicotinamide adenine dinucleotide phosphate-oxidase (NAPDH), 500 μ*M *glucose 6-phosphate, 160 unit/ml glucose 6-phosphate dehydrogenase and 20 m*M *Tris-HCL for 15 minutes at 37°C. Total nitrite was assayed spectrophotometrically at 540 nm using the Griess reaction, as previously described [23]. PGE_2 _production was measured in the culture media using a high-sensitivity enzyme immunoassay according to manufacturer's instructions (R & D systems, Abingdon UK). GAG synthesis was measured in constructs digested overnight at 37°C with 10 U/ml agarase followed by 1 hour at 60°C with 2.8 U/ml papain (both Sigma Chemical Co., Poole, UK) and analyzed with using the DMMB assay as previously described [15]. Total DNA levels were assayed by using the Hoescht dye 33258 in agarose/papain digests [15]. Values for total DNA content remained stable throughout the culture conditions with no significant differences between any treatments. Intracellular and extracellular cGMP was measured in cell lysates and supernatants by ELISA (R & D Systems), in the presence of the pan-PDE inhibitor, 3-isobutyl-1-methylxanthine (IBMX, Sigma-Aldrich). CNP concentration was determined by concentrating the cell culture supernatants on C18 SEP-columns according to the CNP-22 EIA assay kit instructions (Phoenix Pharmaceuticals, Mannheim, Germany). The supernatants (700 ul) were acidified with an equal volume of buffer A, centrifuged and loaded onto columns and equilibrated with buffer A. Each sample was applied to a separate column and allowed to flow through by gravity. Columns were washed twice with 3 ml of buffer A and the CNP eluted with 3 ml of buffer B. The eluate was then evaporated to dryness in a centrifugal speed vacuum and the residue dissolved in 125 μl of EIA assay buffer. CNP concentrations were determined according to the manufacturer's instructions.

### Paraffin embedding, sectioning and detection of natriuretic peptide receptors by immunofluorescence microscopy

Non-diseased (grade 0 to 1) and diseased (grade IV) cartilage specimens from eleven donors (age range 58 to 84 years) were fixed, paraffin-embedded and serially sectioned in a sagittal plane at 10-μm intervals. Tissue morphology was assessed by H&E or Safranin-O fast green staining for highly sulphated, negatively charged GAGs. Slides were analysed on the Leica microscope and images captured with a Hamamatsu digital camera and HiPic32 imaging software. Slides were deparaffinized with xylene, rehydrated through a series of decreasing concentrations of ethanol and washed with tris-buffered saline (TBS) for 5 minutes. Antigen retrieval was achieved with 0.02% HCL at 37°C for 15 minutes, followed by 2.5 mg/mL pepsin in 0.02% HCL at 37°C for 45 minutes. After 3 × TBS washes for 5 minutes, sections were blocked using avidin (10 minutes), biotin (10 minutes) and protein (20 minutes) block (all reagents obtained from DAKO Ely, UK) and incubated with primary antibodies for Npr2 (ab37620) and Npr3 (ab97389, AbCam, Cambridge, UK) overnight at 4°C. Detection was performed by incubating sections with anti-rabbit biotinylated secondary antibodies at a dilution of 1:100 in antibody diluent (all reagents from DAKO) for 1 hour, streptavidin-488 at 1:300 for 30 minutes, 4',6-diamidino-2-phenylindole (DAPI) at 1 μg/mL for 10 minutes, with washing 3 × 5 minutes in TBS after each incubation. Sections were mounted with ProLong antifade (Invitrogen, Paisley, UK), and captured with Image J software by confocal microscopy using 20 × or 63 × objectives (Leica Confocal Microsystem, Milton Keynes, UK). The number of cells positive in the 488 nm channel was counted from a minimum of 30 cells/slide, and the percentage of positively stained cells calculated from the total number of DAPI-positive cells. All images were processed with Adobe Photoshop CS2.

### Western blotting

Western blots were performed to assess total levels of Npr protein in cell lysates generated by digestion of diseased (grade III to IV) and non-diseased (grade 0 to I) cartilage from suitable donors. Human chondrocytes (1 × 10^6^) were lysed in 1% SDS and 40 μg of total protein determined by bicinchoninic acid (BCA) assay was loaded onto SDS-PAGE gels for electrophoresis (Thermo Scientific, Rockford, IL, USA). After semi-dry transfer to nitrocellulose membranes, Nprs were detected by incubation overnight with primary antibodies (1:500) for Npr2 (ab14357, Abcam) or Npr3 (ab97389, Abcam) and detected by IrDye-labelled antibodies for visualisation on the Odyssey system (Li-Cor Biosciences, Lincoln, NE, USA). GAPDH (1:2000) served as an internal control (OAEA00006, Cambridge Biosciences, UK).

### Statistics

For free-swelling studies, data represent the mean and standard error of the mean (SEM) values of up to 25 replicates containing chondrocytes isolated from four to five donors. For the mechanical loading experiments, biochemical and gene-expression data represent the mean and SEM values of up to 13 replicates from three to four separate experiments/donors. Statistical analysis was performed with two-way analysis of variance (ANOVA) and the multiple post hoc Bonferroni-corrected *t-*tests to compare differences between the various treatment groups, as indicated in the figure legend. For gene-expression data, ratio values were log transformed before analysis by two-way ANOVA and post hoc Bonferroni-corrected *t-*test. In all cases, a level of 5% was considered statistically significant (*P *< 0.05).

## Results

### Natriuretic peptide receptor expression increased with age and disease severity

**Figure 1:**
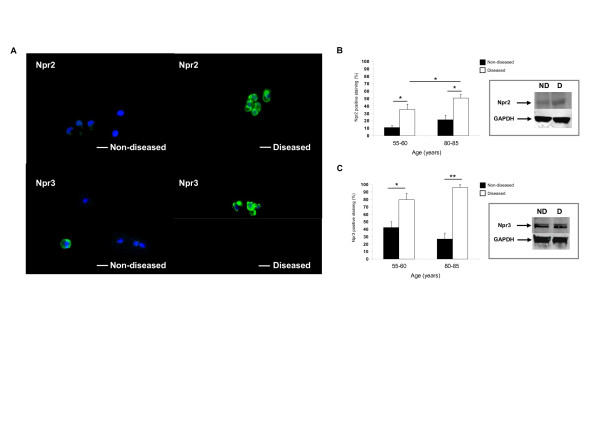
**Comparison of natriuretic peptide receptor 2 (Npr2) and Npr3 expression in non-diseased (grade 0/I) and diseased (grade III to IV) cartilage**. Tissues were taken from donors aged 55 to 60 and 80 to 85 years. Paraffin-embedded sections from a single donor aged 60 years were stained with Npr2 or Npr3 antibodies (green) and examined by immunofluoresence microscopy (**A**). Nuclei (blue) were stained with 4',6-diamidino-2-phenylindole (DAPI). Scale bar represents 10 μM. Negative controls showed no staining (not shown). Inset shows Npr2 (110 kDa) and Npr3 expression (60 kDa) by western blot analysis from the same donor. GAPDH, glyceraldehyde-3-phosphate dehydrogenase.

Immunofluorescence microscopy showed weak localised staining for Npr2 expression in non-diseased cartilage from donors aged between 55 and 60 years (Figure 1A and 1B). In contrast, there was a greater abundance of Npr2 expression in diseased cartilage and the number of positive cells significantly increased with age (*P *< 0.05) (Figure 1B), showing distinct cell membrane localisation (Figure 1B) and expression by western blot analysis (inset, Figure 1B). In non-diseased cartilage, similar levels of Npr3 expression were found for both age groups, with some labelling in the cytoplasm of these cells (Figure 1A). However, Npr3 expression was observed not to be expressed by all cells in older cartilage (Figure 1B). In diseased tissue, the number of Npr3-positive cells significantly increased with age (*P *< 0.01) (Figure 1C), such that greater levels of Npr3 staining was observed in the cell membrane (Figure 1C). Analysis by western blotting showed expression of Npr3 in non-diseased and diseased tissue (inset, Figure 1C). A distinct pattern was therefore observed, with an age-related increase in expression of both Npr2 and Npr3 in diseased cartilage compared to non-diseased tissue.

### Natriuretic peptide receptors mediate a reduction in catabolic effects via cGMP

**Figure 2:**
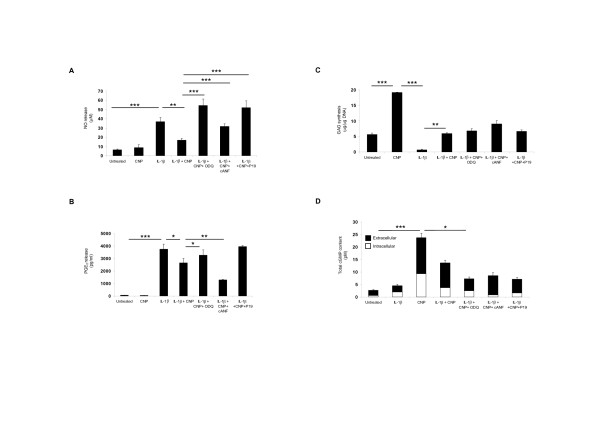
**Effect of pharmacological agents that influence natriuretic peptide and 3,5-cyclic guanosine monophosphate (cGMP) signalling**. Constructs were cultured under free-swelling conditions with 0 or 10 ng/ml IL-1β and/or 100 nM C-type natriuretic peptide (CNP) or 1H-(1,2,4)oxadiazolo-(4, 3-a)quinoxalin-1-one ODQ (5 μM), P19 (0.5 μM) and cANF^4-23 ^(1 μM) on nitric oxide (NO) release (A), prostaglandin E_2 _(PGE_2_) production (B), glycosaminoglycan (GAG) synthesis (C) and total 3,5-cyclic guanosine monophosphate (cGMP) content (D) for 48 hours (*n *= 9 to 25). Asterisks indicate significant comparisons between untreated control samples with IL-1β and/or IL-1 + CNP and/or IL-1β + CNP + cANF^4-23 ^and/or IL-1β + CNP + P19. All other comparisons (not indicated) were not significant.

Treatment of chondrocyte/agarose constructs with IL-1β led to an increase in catabolic effects involving enhanced NO production and PGE_2 _release, and a reduction in GAG synthesis (all *P *< 0.001) (Figure 2A-C). However, co-treatment with CNP prevented IL-1β-induced catabolic effects, resulting in a reduction of NO (*P *< 0.01) (Figure 2A) and PGE_2 _release (*P *< 0.05) (Figure 2B) and restoration of GAG synthesis similar to untreated control values (*P *< 0.01) (Figure 2C). To assess the differential effects of Npr2 or Npr3 signalling, specific peptides that influence the natriuretic peptide receptors were incubated for 48 hours with CNP in IL-1β-treated constructs. Inhibition of the Npr2 receptor with P19 maintained catabolic activities resulting in higher levels of NO and PGE_2 _production when compared to constructs co-cultured with CNP and IL-1β (*P *< 0.001 and *P *< 0.01) (Figure 2A and 2B, respectively). In contrast, treatment with the Npr3 agonist, cANF^4-23 ^had the opposite effect and reduced NO and PGE_2 _release in constructs co-cultured with CNP and IL-1β (*P *< 0.001 and *P *< 0.01) (Figure 2A and 2B, respectively). These findings suggest that the reduction in catabolic activities in response to IL-1β are mediated by both natriuretic peptide receptors.

We next examined the effect of inhibiting sGC with ODQ, as the NO and CNP pathways intersect in the intracellular signalling cascade and are both inducers of cGMP. The effect of ODQ was identical to P19, resulting in high levels of NO and PGE_2 _release (*P *< 0.001 and *P *< 0.05) (Figure 2A and 2B, respectively) without significantly affecting GAG synthesis in constructs co-cultured with CNP and IL-1β. The cytokine did not significantly influence cGMP levels in chondrocyte/agarose constructs (Figure 2D). However, CNP significantly increased cGMP levels in the absence of IL-1β resulting in enhanced GAG synthesis when compared to untreated controls (all *P *< 0.001). Co-stimulation with IL-1β, CNP and the soluble guanylyl cyclase (sGC) inhibitor significantly reduced total cGMP levels when compared to constructs treated with CNP and IL-1β (*P *< 0.05). However, no further significant change was observed with cANF^4-23 ^or P19 on cGMP levels in constructs co-cultured with CNP and IL-1β, resulting in minimal corresponding changes in GAG synthesis.

The relationship between cGMP and GAG synthesis is interesting. The presence of CNP significantly increased cGMP levels and GAG synthesis. However, the anabolic response was inhibited with IL-1β and reversed with CNP in IL-1β-treated constructs. Both the sGC and Npr2 inhibitor but not the Npr3 inhibitor reduced cGMP levels similar to IL-1β-treated constructs. This reduction in cGMP levels by blockade of the natriuretic peptide pathway results in basal levels of GAG synthesis similar to untreated controls. It is evident that CNP acts as a cGMP inducer that mediates anabolic effects via Nprs in chondrocytes.

### Dynamic compression and natriuretic peptide receptors counteracts the pathways induced by IL-1β

**Figure 3:**
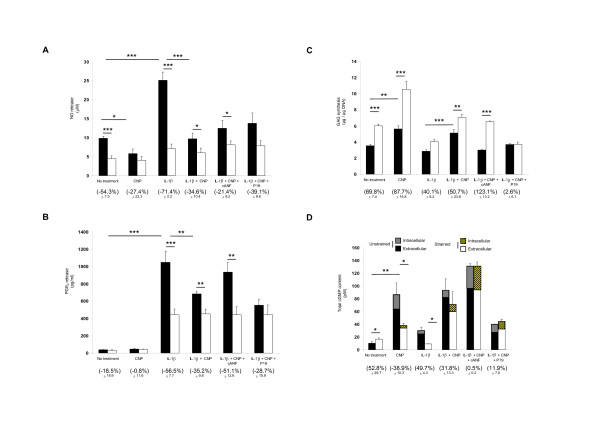
**Effect of dynamic compression and pharmacological agents that influence the natriuretic peptide receptor 2 (Npr2) or Npr3 pathways**. Absolute values for nitric oxide (NO) release (**A**), prostaglandin E_2 _(PGE_2_) production (**B**), glycosaminoglycan (GAG) synthesis (**C**) and total 3,5-cyclic guanosine monophosphate (cGMP) content (**D**). Chondrocyte/agarose constructs were cultured in the presence and absence of 0 or 10 ng/ml IL-1β and/or 100 nM C-type natriuretic peptide CNP or 0.5 μM P19 or 1 μM cANF^4-23 ^for 48 hours. Error bars represent the mean and standard error of the mean (SEM) values of between eight and thirteen replicates from four separate experiments. In addition, the corresponding normalised strained values, presented as a percentage change of the unstrained controls, with SEM values are shown in brackets. Asterisks indicate significant comparisons in unstrained (black bars) and strained (white bars) constructs for the multiple treatment conditions. All other comparisons (not indicated) were not significant.

The effect of CNP and dynamic compression on catabolic (NO and PGE_2_) and anabolic (GAG synthesis, cGMP) activities using peptides, which modulate the Npr2 (P19) and Npr3 pathway (cANF^4-23^), are shown in Figure 3. In the absence and presence of IL-1β, dynamic compression significantly inhibits NO production in chondrocyte/agarose constructs (both *P *< 0.001; Figure 3A). In unstrained constructs, CNP inhibits NO production (*P *< 0.05), but there was no further significant effect with dynamic compression. In the presence of IL-1β, stimulation with CNP and dynamic compression reduced NO release (*P *< 0.05) and the inhibitory effect was not influenced further with cANF^4-23 ^or P19. This resulted in broadly similar percentage change values which ranged between 21 and 39%.

In the absence and presence of CNP, dynamic compression did not significantly influence PGE_2 _release (Figure 3B). In unstrained constructs, IL-1β enhanced PGE_2 _production and this effect was reduced by dynamic compression (both *P *< 0.001) or by stimulation with CNP (*P *< 0.01). Co-stimulation with dynamic compression and the Npr3 agonist reduced PGE_2 _release with a percentage change value broadly similar for constructs cultured with IL-1β and/or CNP. In contrast, the Npr2 inhibitor reduced compression-induced inhibition of PGE_2 _release, implying that this receptor is important in preventing catabolic effects.

Dynamic compression increased GAG synthesis in the presence and absence of CNP (both *P *< 0.001; Figure 3C). The compression-induced stimulatory effect was reduced with IL-1β and restored with CNP in cytokine-treated unstrained (*P *< 0.001) or strained constructs (*P *< 0.01). The presence of cANF^4-23 ^increased compression-induced stimulation of GAG synthesis (*P *< 0.001) with percentage change values greater for CNP-treated constructs when compared to untreated controls (123 and 69%, respectively). However, P19 abolished compression-induced synthesis of GAG in IL-1β-treated constructs, suggesting that the Npr2 receptor is important in mediating anabolic effects in the presence of the cytokine (Figure 3C). Furthermore, we examined the effect of CNP on cGMP levels (Figure 3D). Dynamic compression significantly increased total cGMP content (*P *< 0.05). In unstrained constructs, the presence of CNP and/or IL-1β increased cGMP levels (both *P *< 0.01) and the response was reduced with dynamic compression (*P *< 0.05). The presence of cANF but not P19 enhanced cGMP levels and the response was not influenced further with dynamic compression.

### Dynamic compression increased CNP levels and natriuretic peptide receptor expression which mediate an anabolic response

**Figure 4:**
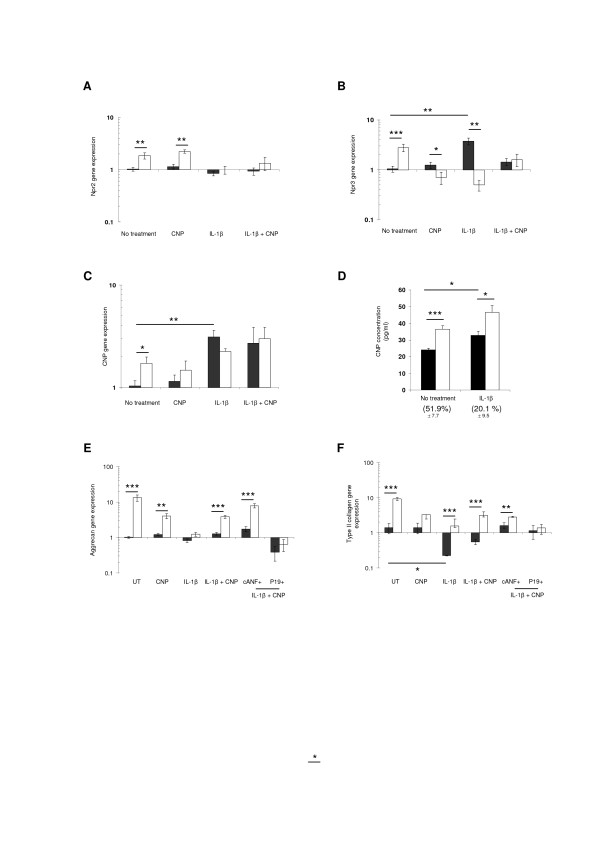
**Effect of C-type natriuretic peptide (CNP) and dynamic compression on natriuretic peptide receptors (Nprs) and CNP levels**. Npr2 (**A**), Npr3 (**B**) CNP (**C**) and CNP concentration (**D**), aggrecan (**E**) and type II collagen gene expression (**F**). Constructs were cultured in the presence and absence of 0 or 10 ng/ml IL-1β and/or 100 nM CNP or 0.5 μM P19 or 1 μM cANF^4-23 ^6 hours. Error bars represent the mean and standard error of the mean (SEM) values of eight to thirteen replicates from four separate experiments. Asterisks indicate significant comparisons in unstrained (black bars) and strained (white bars) constructs for the multiple treatment conditions. All other comparisons (not indicated) were not significant. For CNP concentration, numbers in brackets indicates the percentage change from unstrained control values.

Since mechanical loading enhanced the pathways induced by CNP, it was important to examine whether dynamic compression could influence gene expression of Nprs and CNP levels (Figure 4). In the presence and absence of CNP, dynamic compression significantly increased Npr2 gene expression (both *P *< 0.01) (Figure 4A). Dynamic compression increased Npr2 expression in the absence and presence of CNP (both *P *< 0.01; Figure 4A, but not with IL-1β. In untreated constructs, dynamic compression increased Npr3 expression (*P *< 0.001) and the response was reduced with CNP (*P *< 0.05) (Figure 4B). In unstrained constructs, IL-1β significantly increased Npr3 expression (*P *< 0.01) and this effect was inhibited with dynamic compression (*P *< 0.001). Either dynamic compression (*P *< 0.05) or the presence of IL-1β significantly increased CNP gene expression (*P *< 0.01; Figure 4C). The stimulatory effect was not influenced further with CNP and/or IL-1β and/or stimulation with dynamic compression. In addition, CNP concentration was significantly enhanced with dynamic compression (*P *< 0.001) and/or IL-1β (*P *< 0.05) (Figure 4D). This led to the induction of aggrecan and collagen type II gene expression by dynamic compression in constructs cultured with CNP or cANF but not with P19 (Figure 4E, F).

**Figure 5:**
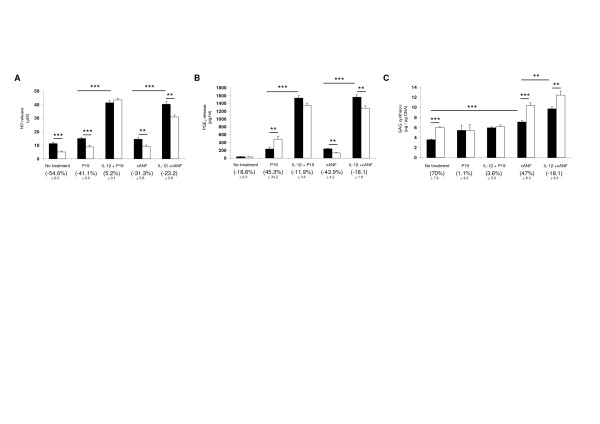
**Effect of dynamic compression and pharmacological agents that influence the natriuretic peptide receptor 2 (Npr2) or Npr3 pathways**. Absolute values for nitric oxide (NO) release (**A**), prostaglandin E_2 _(PGE_2_) production (**B**) and glycosaminoglycan (GAG) synthesis (**C**). Constructs were cultured in the presence and absence of 0.5 μM P19 or 1 μM cANF^4-23 ^for 48 hours. Error bars represent the mean and standard error of the mean (SEM) values of eight replicates from three separate experiments. In addition, the corresponding normalised strained values, presented as a percentage change of the unstrained controls, with SEM values are shown in brackets. Asterisks indicate significant comparisons in unstrained (black bars) and strained (white bars) constructs for the multiple treatment conditions. All other comparisons (not indicated) were not significant.

We next examined whether mechanical loading could influence endogenous CNP signalling. P19 and cANF^4-23 ^did not influence the inhibitory effect of NO release in response to dynamic compression (Figure 5A). However, in the presence of IL-1β, the inhibitory response was abolished with P19 but not with cANF. In addition, compression did not influence PGE_2 _release in the absence of P19 but the levels were increased with P19 or reduced with cANF^4-23 ^(both *P *< 0.01) (Figure 5B). IL-1β enhanced PGE_2 _release and stimulation with dynamic compression reduced PGE_2 _release with cANF (*P *< 0.01) but not with P19. In unstrained constructs, GAG synthesis was enhanced with cANF^4-23 ^(both *P *< 0.001) (Figure 5C). However, the compression-induced stimulatory effect on GAG synthesis was abolished with P19 and significantly enhanced with cANF^4-22 ^in the absence (*P *< 0.001) and presence of IL-1β (*P *< 0.01). The data demonstrate that mechanical loading-induced CNP is protective and stimulates an anabolic response by altering the expression of the Nprs and CNP levels.

## Discussion

The signalling pathways leading to the cytoprotective effects of CNP are currently unclear. In this study, we examined the mechanisms by which CNP leads to a reduction of catabolic activities induced by IL-1β. CNP predominantly activates Npr2, which underlies guanylyl cyclase activity and mediates several cellsignalling effects through the synthesis of cGMP. In addition, CNP binds to Npr3, which does not possess guanylate cyclase functionality and until recently, was thought to be devoid of any natriuretic peptide signalling capacity. Although several studies have suggested a role for Npr2 signalling in cartilage and bone, recent evidence demonstrates that the Gi/o binding domain in Npr3 activates ERK 1/2, which is important in endothelial and vascular smooth muscle cell proliferation and cardiovascular homeostasis [4,8,14]. Both Npr2 and Npr3 play a role in cartilage and bone homeostasis in knockout mouse models, although the precise mechanisms remain unclear [5,7]. Furthermore, in rat osteoblasts, expression of Nprs was shown to change with age with a shift in expression from Npr2 to Npr3 in older rats [13].

Given the role of age in the pathogenesis of OA, we were interested to observe any disease and/or age-related effects of Nprs and compare expression at the mRNA and protein levels in non-diseased and OA-affected cartilage isolated from donors undergoing total knee arthroplasty with an age range of 55 to 85 years. The present study confirmed dominant expression of Nprs in diseased cartilage, when compared to non-diseased tissue from the same donor. In addition, there was significant upregulation of both receptors in older cartilage (age 70 years), such that Npr3 expression appeared to be greater in abundance than Npr2 in tissue taken from the same donor. However, we did not compare Npr expression in young cartilage (age range from 30 to 39 or 40 to 49 years) isolated from a healthy joint and this merits further investigation. In addition, Npr3 but not Npr2 mRNA levels were significantly higher with IL-1β when compared to untreated controls. Given the chondroprotective nature of CNP, the present study suggests that OA chondrocytes upregulate natriuretic peptide signalling as a consequence of the pro-inflammatory process, possibly as a means to restrict disease progression. Furthermore, Nprs appear to be linked with the age and/or the disease process enabling osteoarthritic tissue to retain its ability to respond to CNP if administered as a therapeutic intervention. Indeed, the next step in this research is to test this hypothesis in an *in vivo *mouse model, which represents early-stage OA, enabling exploitation of transgenic animals with deficiencies in CNP signalling (for example, tissue-specific Npr2^-/-^, Npr3^-/-^) in the absence and presence of agents that stimulate the Npr pathways. For example, mice lacking Npr3 facilitate subtype switching during differentiation from proliferating to hypertrophic chondrocytes in the growth plate of the fetal mouse tibia [24]. However, no studies have examined whether mice lacking Npr3 stimulate pro-inflammatory cytokines leading to both a loss of cGMP signalling and the chondroprotective effects in cartilage. Thus, the actions of pro-inflammatory cytokines on natriuretic peptide signalling in chondrocyte functions warrant further examination.

**Figure 6:**
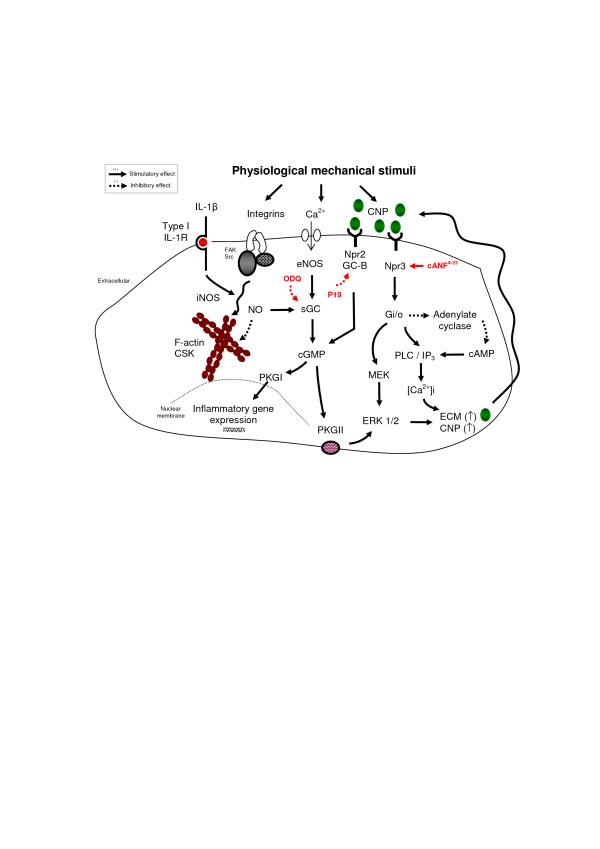
**Proposed signalling interactions between C-type natriuretic peptide (CNP) and mechanical loading in chondrocytes**. CNP primarily activates natriuretic peptide (Npr)2 which underlies guanylyl cyclase (GC) activity and mediates several cell-signalling effects through the synthesis of 3,5-cyclic guanosine monophosphate (cGMP) and membrane-bound cyclic GMP-dependent protein kinase II (PKGII). It has been reported that the Gi/o binding domain in Npr3 activates extracellular-signal-regulated kinase 1/2 (ERK 1/2) or the phospholipase C (PLC) and inosital triphosphate (IP_3_) pathway via adenylate cyclase/cAMP inhibition. PLC activation and IP_3 _generation is well known to contribute to Ca^2+ ^release from intracellular stores. This pathway supports cartilage homeostasis by mediating the anabolic effects involved in mechanotransduction. Furthermore, mechanical signals stimulate Npr expression and CNP levels, which mediates anabolic effects involving extracellular matrix (ECM) synthesis. In contrast, IL-1β stimulates catabolic activities via inducible nitric oxide synthase (iNOS) and nitric oxide (NO)/sGC/cGMP levels. The IL-1β-induced catabolic response could be inhibited by mechanosensitive CNP or Ca^2+ ^release through stretch-activated ion channels or integrin-mediated signals involving F-actin/cytoskeletal reorganisation.

We investigated whether Npr pathways have differential or overlapping protective roles in maintaining cartilage homeostasis. This was achieved by utilising two pharmacological agents that specifically inhibit Npr2 (P19) or activate Npr3 (cANF^4-23^). In the present study, the Npr2 antagonist prevented the protective effects of CNP on catabolic activities by maintaining high levels of NO and PGE_2 _release in IL-1β-treated constructs, whilst not affecting GAG synthesis. The findings are in agreement with a recent study, which reported that CNP could signal through Npr3 and exert an anabolic response in endothelial cells [14]. As shown in the schematic in Figure 6, Npr3 mediates several anabolic effects linked to cartilage homeostasis involving adenylate cyclase inhibition and stimulation of the G1 protein, phospholipase C (PLC) and inosital triphosphate (IP_3_) pathway [13,25]. In chondrocytes, PLC activation and IP_3 _generation contribute to Ca^2+ ^release from intracellular stores, which is known to support cartilage homeostasis by mediating the anabolic effects involved in mechanotransduction [26-30]. These findings suggest that Npr3 could influence adenylate cyclase/cAMP signalling, thereby altering the relative balance between the cyclic nucleotides and downstream signal transduction events (Figure 6).

There was strong parallelism in the actions of CNP on cGMP induction, resulting in enhanced GAG synthesis and reduction of catabolic effects induced by IL-1β. Both NO and CNP signalling lead to the synthesis of cGMP, indicating there is cross-talk between IL-1β/NO/sGC/cGMP-mediated catabolism and CNP-mediated chondroprotection [31]. It has previously been reported that the temporal differences in NO/cGMP signalling leads to differential, biphasic effects [32]. Indeed, in a previous study, catabolic events involving NO release are characterized by an initial spike in cGMP levels, in contrast to anabolic signals, which are linked to low, more sustained cGMP turnover [32]. A similar mechanism may explain the parallel involvement of cGMP in our model, whereby the IL-1β-induced NO/sGC/cGMP signalling route mediates catabolic activities in contrast to the Npr2/GC-B/cGMP/PKGII pathway, which is anabolic. Interestingly, in our experiments we showed the presence of extracellular cGMP, exceeding intracellular concentrations, which were both reduced with the sGC inhibitor, ODQ. These results are consistent with studies in smooth muscle cells or brain astrocytes, which demonstrates a similar pattern of extracellular cGMP levels when compared to intracellular concentrations reported to be less than 20 pM [33,34]. Recent studies suggest that members of the ATP-binding cassette transport system, in particular multidrug resistance protein 5 (MRP5), regulate extrusion of cGMP and prevent hyaluronan export in fibroblasts [35,36]. The biological significance of cGMP transporters in chondrocytes have yet to be clarified. In contrast, there is ample evidence that shows the clinical importance of hyaluronan in OA treatment [37]. A recent study analysed the effects of enhanced cGMP levels with PDE5 inhibitors demonstrating prevention of HA over-production and proteoglycan/collagen loss in IL-1α-treated cartilage explants [38]. However, regulation of HA export through MRP5 and CNP-linked Npr2/cGMP signalling has not been previously described in human cartilage and merits further examination.

To test the hypothesis that mechanical loading interferes with the signals induced by natriuretic peptides, we incubated constructs with agonists or antagonists of the Npr2 and Npr3 pathways to show that biomechanical and natriuretic peptide signals stabilise cartilage homeostasis. In the presence of CNP, specific inhibition of Npr2 abolished the anabolic response to mechanical loading and prevented loading-induced inhibition of NO and PGE_2 _release. In contrast, the presence of the Npr3 agonist had the opposite effect and significantly increased GAG synthesis and cGMP levels in response to mechanical loading and reduced NO and PGE_2 _release comparable to control samples. cGMP levels are a good index of CNP/Npr2 signalling, particularly since the increase in cGMP in response to CNP is blocked with P19. The Nprs are therefore critical mediators necessary for anabolic signalling in response to CNP and mechanical loading. In a previous study, we described PKGII as the principal mediator of cGMP signals, thereby implying that the Npr2/sGC/cGMP/PKGII pathway has a positive role in cartilage homeostasis [3]. Indeed, mechanical strain was reported to significantly increase cGMP synthesis in mouse podocytes and induce subsequent anabolic activities [39]. This is in agreement with the present study, which showed that signals involving compression or CNP are cGMP inducers leading to anabolic events.

Furthermore, the present data demonstrate differential effects of Nprs on NO and PGE_2 _release, implicating cross-talk with other pathways involving IL-1β. For example, mechanical loading is known to influence several overlapping genes that influence the NFκB and mitogen-activated protein kinase (MAPK) pathways leading to downregulation of NO and PGE_2 _production [39-43]. However, the time course of phosphorylation events in response to CNP, IL-1β and dynamic compression have not been presented in the current study. A limitation of the *ex vivo *3D/bioreactor model, are the difficulties associated with isolating cell lysates from pooled constructs, which are largely agarose gel and associated media-serum contaminant rather than being cell derived. Compression, IL-1β or CNP could affect ERK phosphorylation, but may also have action on other MAPK signalling pathways, which alter their activation state in a time-dependent manner. Indeed, the presence of the critical mechanosenstive integrins and cytoskeleton will additionally counteract IL-1β-induced catabolic activities [43-45]. Indeed, biomechanical signals have been shown to upregulate both cGMP and cAMP production in osteoblasts or PLC signalling in chondrocytes, which are critical factors involved in the Npr2 and Npr3 pathways [29,46-48]. We therefore asked the question whether mechanical loading could directly influence expression of Nprs, providing an autocrine/paracrine mechanism for the differential effects of CNP (Figure 6). Indeed, the present study confirmed CNP levels and Npr expression were increased with mechanical loading in chondrocyte/agarose constructs, demonstrating an endogenous role of these factors. Our observations are in agreement with a previous study, which demonstrates the induction of CNP in response to shear stress in vascular cells [49]. Furthermore, the presence of IL-1β or TNFα significantly induced CNP expression in endothelial cells, supporting an endogenous role for natriuretic peptide signalling in the prevention of pro-inflammatory induced signals [50].

In summary, Nprs are influenced by CNP, IL-1β, age, disease severity and mechanical loading. The differential effects of CNP are dependent on the combination of factors that influence cartilage homeostasis, leading to the activation of multiple, temporal events, which affect downstream pathways. Biomechanical signals stimulate natriuretic peptide signalling, which is protective and maintains cartilage health. Future studies are needed to confirm the beneficial effects of CNP and mechanical signals in an *in vivo *mouse model that represents early stage OA.

## Conclusions

CNP plays a critical role in the development and regulation of articular cartilage by promoting extracellular matrix production and chondrocyte proliferation. Our previous studies demonstrate that CNP acts to inhibit catabolic signals in response to IL-1β, and that these effects are synergistic with the protective stimuli induced by mechanical loading. The present study demonstrates that endogenous CNP/Npr2/cGMP signalling route mediates anabolic events and prevents catabolic activities induced by IL-1β. Stimulation with biomechanical signals and natriuretic peptide signals further augments the anabolic response, resulting in a reduction of catabolic events mediated by the Npr2/GC/cGMP route. Therapeutic application of CNP, or interventions targeted to Nprs to mimic the actions of CNP should, therefore, be considered to speed up repair mechanisms and stabilise cartilage homeostasis in osteoarthritic conditions.

## Abbreviations

ANOVA: analysis of variance; BCA: bicinchoninic acid; CGI: cGMP-regulated ion channels; cGMP: 3,5-cyclic guanosine monophosphate; CNP: C-type natriuretic peptide; C_t_: cycle threshold; DAPI: 4',6-diamidino-2-phenylindole; DMEM: Dulbecco Modified Eagle Medium; EBSS: Earle Balanced Salt Solutions; ERK: extracellular signal-regulated kinase; FCS: fetal calf serum; GAG: glycosaminoglycan; GAPDH: glyceraldehyde-3-phosphate dehydrogenase; H&E: hematoxylin & eosin; ICRS: International Cartilage Repair Society; IL-1β: interleukin-1β; IP_3_: inosital triphosphate; MAPK: mitogen-activated protein kinase; MRP5: multidrug resistance protein 5; NAPDH: nicotinamide adenine dinucleotide phosphate-oxidase; NFκB: nuclear factor κB; NO: nitric oxide; Npr: natriuretic peptide receptor; OA: osteoarthritis; ODQ: 1H-(1,2,4)oxadiazolo-(4, 3-a)quinoxalin-1-one; PDE: cGMP-regulated phosphodiesterase; PGE_2_: prostaglandin E_2; _PLC: phospholipase C; qPCR: quantitative polymerase chain reaction; SEM: standard error of the mean; sGC: soluble guanylyl cyclase; TBS: tris-buffered saline.

## Competing interests

The authors declare that they have no competing interests. The work was supported by the AO Research Fund of the AO Foundation (S-09-83C) and Arthritis Research UK (19646).

## Authors' contributions

NP, NS, AM, AH and TC carried out the experiments and analysis, participated in the experimental design, data analysis and manuscript drafting. AH, PA, DB, MR and DS participated in the experimental design, data analysis, and manuscript drafting. All authors read and approved the final manuscript.
